# Characterization of porcine sapelovirus prevalent in western Jiangxi, China

**DOI:** 10.1186/s12917-021-02979-7

**Published:** 2021-08-14

**Authors:** Taotao Yang, Lingqian Zhang, Yingmei Lu, Minhong Guo, Zhibang Zhang, Anqi Lin

**Affiliations:** College of Life Sciences and Resource Environment, Yichun University, Yichun, 336000 Jiangxi China

**Keywords:** *Porcine sapelovirus*, Prevalence, Pigs, Jiangxi

## Abstract

**Background:**

*Porcine sapelovirus* (PSV) infection can lead severe polioencephalomyelitis with high morbidity and mortality, which result in significant economic losses. Infection with the PSV is believed to be common yet limited information is available on the prevalence and molecular characterization of PSV in China. Therefore, the objective of this study was to characterize the prevalence and genome of PSV strains identified in the western Jiangxi province of China.

**Results:**

A high specificity and sensitivity SYBR Green I-based RT-PCR method for PSV detection was developed. Two hundred and ninety four fecal samples were collected from December 2018 to March 2019 in 4 farms. An overall PSV-positivity rate of 11.22% (33/294) was detected with the real-time RT-PCR method, and a high infection rate and viral load of PSV were found in nursery pigs. In total, complete *VP1* gene sequences of 11 PSV strains (PSV-YCs) were obtained. Homology comparisons of the *VP1* gene of the 11 PSV-YCs with previously reported PSVs revealed nucleotide sequence identities ranging from 63% to 96.8%, and deduced amino acid sequence identities from 61.4% to 99.7%. Phylogenetic analyses based on the *VP1* gene exhibited 2 main clades corresponding to PSV-1 and PSV-2, and all PSV-YCs prevalent in western Jiangxi belonged to the traditional genotype (PSV-1). In addition, the pairwise distances of *VP1* gene sequences between PSV-YCs ranged from 0.009 to 0.198, which indicating that substantial genetic diversity among the PSVs in western Jiangxi.

**Conclusions:**

To the authors’ knowledge, this is the first description of PSV in the Jiangxi province pig herds in China, and it is crucial to understand the epidemiology of the viruses in China. The results also provide an important theoretical foundation for diagnosis and early warning of epidemic diseases caused by PSVs prevailing in this region.

**Supplementary Information:**

The online version contains supplementary material available at 10.1186/s12917-021-02979-7.

## Background

Taxonomically, Porcine sapelovirus (PSV), also *Sapelovirus A*, together with *Sapelovirus B* belong to the genus Sapelovirus within the family *Picornaviridae* [1]. The PSV genome consists of a positive-sense, single-stranded RNA of ~7.5 kb, and comprises the typical picornavirus genome organization; 5′-UTR^IRES-IV^-[L/1A-1B-1C- 1D/2A^pro^-2B-2C/3A-3B-3C-3D]-3′-UTR. The 1A-1D genes encode four structural proteins (also named *VP1-4*) to compose virus capsid, and the other genes encode nonstructural proteins [2].

Domestic pigs and wild boars are the only known hosts, and PSV infection can lead severe polioencephalomyelitis with high morbidity and mortality, which result in significant economic losses [3]. However, PSV infections are always subclinical or only involve a series mild symptoms, including spinal cord damage, inappetence, diarrhea and breathless [4, 5]. The disease of PSV infection was first found in Britain in the 1960s [6]. Afterwards, wide infections of PSV in domestic and wild pigs have been reported in many other countries, including Spain, Hungary, Czech Republic, Brazil, China, and South Korea [2, 4, 7–10]. In the 10th Report of the International Committee on Taxonomy of Viruses (ICTV), PSV only comprised a single genotype. With the recent use of viral metagenomics tool, a novel member of PSV genotypes has been discovered in Hungary [11].

Despite several investigations have documented PSV infection in China, knowledge of the molecular prevalence of PSV in many areas of China is still unclear so far [4, 5, 12, 13]. In the present study, for the first time, a novel SYBR Green I-based real-time RT-PCR assay was developed to investigate the prevalence of PSV in western Jiangxi, China. Moreover, genetic and phylogenetic analyses based on *VP1* gene were performed to determine their molecular and evolutionary characterization.

## Results

### PSV real-time PCR performance and prevalence

A linear regression relationship of the standard curves was observed with a correlation coefficient (R^2^) of 0.9996, a slope of -5.1538, and an intercept of 50.716 (Fig. S1a). Specificity of the reaction was confirmed by a distinct melting temperature (Tm) of 84.82 (Fig. S1b), and there was no positive signal when testing cross reactivity against common porcine viral pathogens, including CSFV, PRRSV, PRV, PEDV, PTV, and JEV (Fig. S1c). The sensitivity of real-time PCR method was determined to be 5.22×10^2^ copies/μL (Fig. S1d), which 10 times more sensitive than the conventional RT-PCR (Fig. S1e).

PSV was detected in all 4 commercial farms, representing an overall PSV-positivity rate of 11.22% (33/294). Specifically, PSV was identified in 30.36% (17/56) of the nursery pigs, in 8.82% (12/136) of the fattening pigs, and in 7.27% (4/55) of the adult pigs (Table 1). While no positive sample was detected from suckling pigs. Thus, high PSV prevalence was found in nursery pigs, which was significantly (*P* < 0.01) higher than that in adult pigs, suckling and fattening pigs (Table 1). Fecal samples positive for PSV had CT values of 25.25-38.57, corresponding to the amount of PSV RNA ranged from 6.81×10^5^ to 2.62×10^8^ genomic copies per mL fecal supernatant. Notably, the high PSV viral loads (3.25±0.18, log10 copies/μL) were also identified in the age group of nursery pigs (Table 1).

**Table 1:** Summary of the history (age, number of farms) and prevalence of PSV in western Jiangxi, China

Age group	No. of farms	No. of samples	PSV-positive rate	Viral loads (x̅±SE, log10 copies/μL)
Suckling pigs (5-28 days)	3	47	0% (0/47)	NA
Nursery pigs (29-56 days)	3	56	30.36% (17/56)	3.25±0.18
Fattening pigs (8-25 weeks)	4	136	8.82% (12/136)	2.88±0.10
Adult pigs (>25 weeks)	3	55	7.27% (4/55)	2.83±0.06
Total	4	294	11.22% (33/294)	3.07±0.11

*NA* not available

### Molecular characteristics of PSV genome

**Fig. 1:**
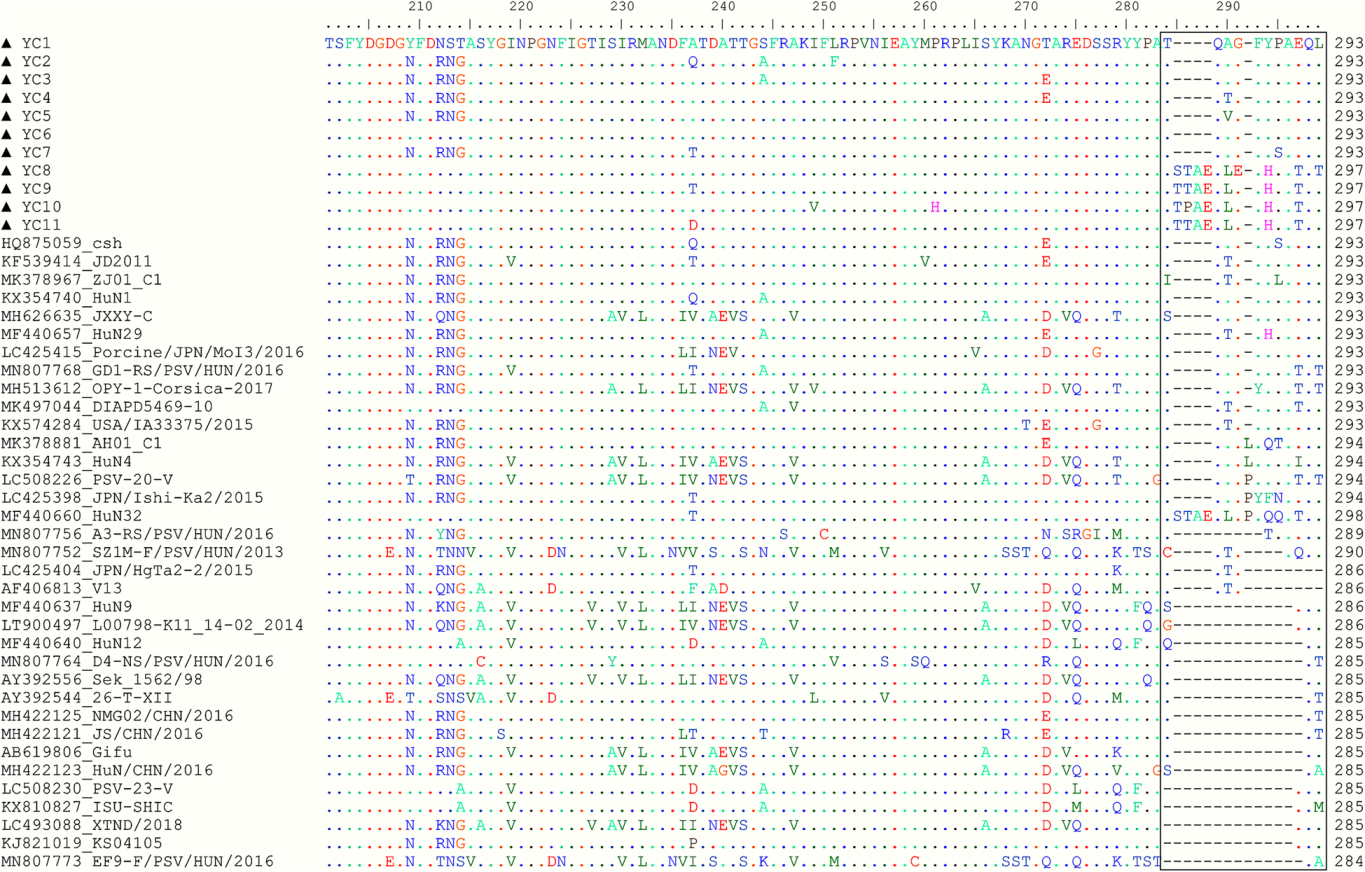
Alignment of the partial amino acid sequences of PSV strains. Alignment of the amino acid sequences near the 3′ end of the *VP1* gene. Sequences in boxes show the hypervariable region in the C-terminus of *VP1*. The strains identified in the present study are indicated by ▲; (-), missing sequences

Eleven complete *VP1* gene sequences were obtained from the 33 PSV-positive samples. Subsequently, the obtained PSV sequences (designated as YC1-11) were submitted to the GenBank database (accession nos. MW411420-MW411430). Through alignment of the coding sequences of the 11 PSV-YC strains with those of other known PSVs, the *VP1* gene of seven PSV-YC strains (YC1-7) containing 897 nucleotides and encoding a 293 amino acid (AA) protein. The other four PSV-YC strains, YC8-11, contained twelve additional nucleotides, corresponding to four additional AAs (S/T)(T/P)AE inserted between AA 283 and 284 of the *VP1* as compared with all other known PSV strains exhibiting the most common genome organization (Fig. 1). Homology comparison of the *VP1* genes of the 11 PSV-YC strains with those of other PSV strains from GenBank revealed nucleotide and deduced amino acid sequence identities ranging from 63% to 96.8% and from 61.4% to 99.7%, respectively. In addition, PSV-YCs had sequence identities of 79.9–99% (nucleotides) and 88.3–99.7% (AAs) with each other.

### Phylogenetic analysis of PSV

**Fig. 2:**
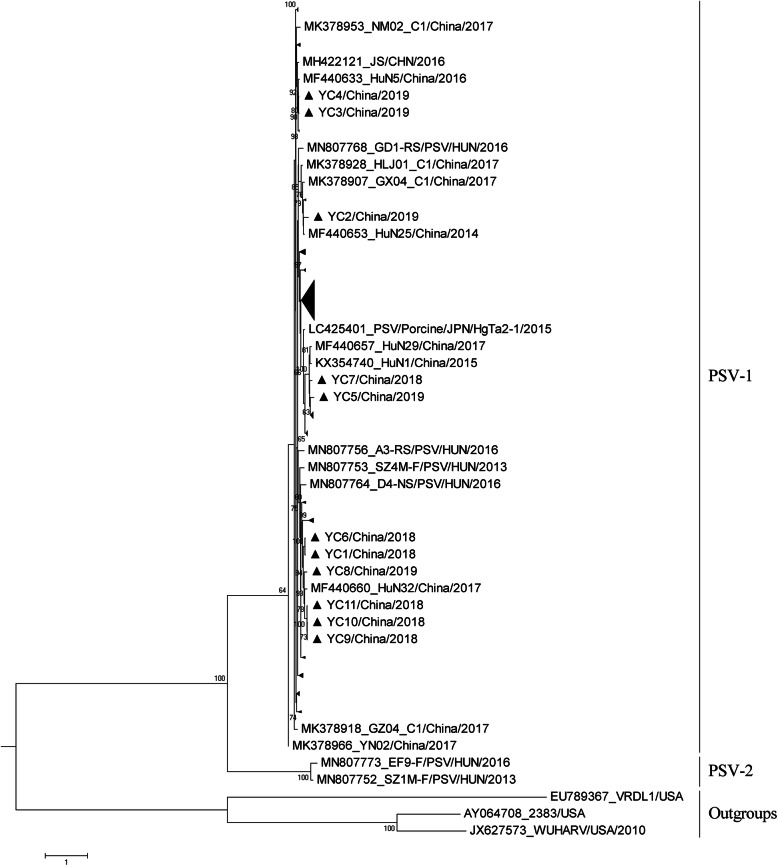
Evolutionary relationships among PSVs. Phylogenetic tree based on the nucleotide sequences of *VP1* gene. The evolutionary history was inferred using the ML method with the GTR model with gamma-distributed rates and proportion of invariant sites (G+I) in PhyML 3.0. The percentage of replicates in which the associated virus clustered together in the bootstrap test (100 replicates) is shown next to the branches (only values >60 % are shown) in the tree. The scale bar indicates nucleotide substitutions per site. Simian sapelovirus (SSV)-1 2383 strain, SSV-2 VRDL strain and SSV-3 WUHARV strain were used as outgroups. The strains isolated in the present study are identified by ▲

Phylogenetic analysis was conducted based on the multiple sequence alignment of the complete *VP1* gene sequences of different PSV, which including the 11 PSV-YCs characterized in the present study and 101 additional PSV sequences obtained from GenBank database (Table S1). Phylogenetic analysis of *VP1* gene sequences showed that all PSVs were separated into two distinct clades, corresponding to two proposed genotypes (designated as PSV-1 and PSV-2) with high bootstrap values within the genus Sapelovirus A (Fig. 2). All the PSV-YC strains fell into PSV-1 along with most of the previously reported PSVs, and two Hungarian strains SZ1M-F/PSV/HUN/2013 and EF9-F/PSV/HUN/2016 were separated into PSV-2 (Fig. 2). Additionally, the pairwise distances of *VP1* gene sequences between PSV-YCs ranged from 0.009 to 0.198, revealing the high genetic diversity among the most prevalent PSVs in pig populations of western Jiangxi, China (Table 2).

**Table 2:** The estimates of evolutionary divergence over sequence pairs between PSV-YCs identified by the neighbor-joining method, which include *VP1* gene sequence of 11 PSVs

Genetic distance between PSV-YCs (mean ± SE)											
	PSV-YC1	PSV-YC2	PSV-YC3	PSV-YC4	PSV-YC5	PSV-YC6	PSV-YC7	PSV-YC8	PSV-YC9	PSV-YC10	PSV-YC11
PSV-YC1		-	-	-	-	-	-	-	-	-	-
PSV-YC2	0.163±0.012		-	-	-	-	-	-	-	-	-
PSV-YC3	0.151±0.011	0.150±0.012		-	-	-	-	-	-	-	-
PSV-YC4	0.149±0.011	0.150±0.012	0.016±0.004		-	-	-	-	-	-	-
PSV-YC5	0.180±0.012	0.176±0.013	0.162±0.011	0.158±0.011		-	-	-	-	-	-
PSV-YC6	0.017±0.004	0.163±0.012	0.148±0.011	0.147±0.011	0.184±0.012		-	-	-	-	-
PSV-YC7	0.168±0.012	0.180±0.013	0.152±0.012	0.147±0.011	0.114±0.011	0.170±0.012		-	-	-	-
PSV-YC8	0.100±0.010	0.189±0.013	0.164±0.012	0.167±0.012	0.190±0.013	0.105±0.011	0.197±0.013		-	-	-
PSV-YC9	0.107±0.011	0.177±0.013	0.155±0.012	0.154±0.011	0.193±0.012	0.097±0.010	0.171±0.012	0.108±0.010		-	-
PSV-YC10	0.109±0.010	0.181±0.013	0.159±0.011	0.158±0.011	0.198±0.012	0.099±0.010	0.173±0.012	0.111±0.010	0.009±0.003		-
PSV-YC11	0.109±0.011	0.177±0.012	0.155±0.012	0.154±0.011	0.195±0.012	0.099±0.010	0.175±0.012	0.108±0.010	0.011±0.004	0.016±0.004	

The numbers of base differences per site from averaging over all sequence pairs between PSV-YCs are shown. Standard error (SE) estimates are shown and were obtained by a bootstrap procedure (1000 replicates). The analyses involved 11 nucleotide sequences were conducted using the p-distance model. All positions containing gaps and missing data were eliminated. There were a total of 879 positions in the final dataset. Evolutionary analyses were conducted in MEGA6

## Discussion

Although PSV is ubiquitous and is associated with a variety of clinical symptoms in domestic and wild pigs, PSVs infecting swine are often ignored for their subclinical manifestations and co-infections with other pathogens [9, 10]. Therefore, it is necessary to develop a convenient, sensitive and accurate detection method for PSV early warning and rapid diagnosis. The PSV SYBR Green I-based real-time RT-PCR method developed in the present study had the advantages of high specificity and sensitivity. The minimum detection limit of sensitivity test showed 522 copies/μl located at same order of magnitude with the Taqman-based real-time RT-PCR method established by Chen J et al [14]. Specially, the PSV SYBR Green I-based real-time RT-PCR method in this study has high cost-efficient compared to the TaqMan-based method, which can achieving wide application with high standards and low cost. Thus, our method can be applied for clinical monitoring and diagnosis of PSV infection.

The high PSV prevalence was found in nursery pigs compared to sporadically in adult and fattening pigs. Meanwhile, higher viral loads were also found in the age group of nursery pigs. While no positive sample was detected in suckling pigs, which possibly associated with the presence of maternal antibodies in piglets. Thus, nursery pigs become the susceptible populations of PSV infection with decreasing of maternal antibodies of suckling pigs as age increasing. PSV widely prevalent in pigs of all age groups have been reported in previous study, especially high prevalence in nursery and fattening pigs, which is somewhat different from the results in the present study [5]. Therefore, we speculated that multiple factors, including the conformation or structure of farm, the health and neutralizing antibody level of pigs, are likely responsible for the differences.

The capsids of picornaviruses are composed of four structural proteins (*VP1*–*4*), and outer surface structure of virion, as also immunodominant, is mainly determined by the spatial folding and mutual extension of *VP1*, *VP2* and *VP3* [15]. Generally, C-terminus of *VP1–3* is located on the outer surface of the virion, while the N-terminus lying on the inner surface of the virion [16]. Thus, amino acid mutations in the C-terminus of *VP1–3* were suggested to influence the antigenicity of picornaviruses. In the present study, four AAs (S/T)(T/P)AE insertion near the C-terminus of *VP1* were found in four PSV-YCs, which was consistent with our previous report. Exposed antigenic sites in the C-terminus of *VP1* have been proved induce neutralizing antibodies in many picornaviruses [17–21]. Therefore, mutations of the C-terminus of the PSV *VP1* possibly are associated with the escape from host immune response. However, the function and specific biological characteristics of those mutations requires more research. Notably, evolutionary analysis revealed the PSV strains with (S/T)(T/P)AE amino acid residues insertion in the C-terminus of *VP1* sharing a close genetic relationship, which indicated those of amino acid residues insertion might be used as molecular marker for a cluster of PSV epidemic strains.

PSV have long been contained only one genotype, recently, a novel PSV strain (SZ1M-F/PSV/HUN2013, MN80775) was reported in pigs of Hungary, which represent a novel genotype of PSV (designed as PSV-2 in the present study) [11] . In this study, phylogenetic analysis of all available *VP1* gene sequences of PSVs showed the PSV-YCs prevalent in western Jiangxi belonged to the traditional genotype (PSV-1). Notably, PSV-YCs shared the closest genetic relationship with the strains found in Hunan Province of China, indicating that the strains prevalent in local areas or neighboring provinces likely evolved from common ancestors. Meanwhile, genetic divergence also exists within the PSVs of western Jiangxi, China, even on a same farm, which was consistent with previous report in other regions [5].

## Conclusion

In conclusion, we developed a high specificity and sensitivity SYBR Green I-based RT-PCR method for PSV detection. A high infection rate and viral load of PSV were found in nursery pigs in western Jiangxi, China. In addition, PSV strains prevalent in this area showed a close evolutionary relationship with the PSVs previously reported in China. Meanwhile, our data revealed substantial genetic diversity among the PSVs in western Jiangxi, China.

## Materials and methods

### Samples and viral strains

During December 2018 to March 2019, 294 fecal samples were collected from healthy pigs across 4 farms in the western Jiangxi province, China. The age of the pigs sampled ranged from piglets to adult pigs (Table 1). Samples were transported to the laboratory at 4 ^о^C and stored at -80 ^о^C until required.

PSV (HuN1 strain, GenBank # : KX354740), pseudorabies virus (PRV, XiangA strain, GenBank # : KP710981), Japanese encephalitis virus (JEV, HNML1 strain, GenBank # : KX774636), and porcine teschovirus (PTV, HuN1 strain, GenBank # : MF170905) were stored in our laboratory at -80 ^о^C. Classical swine fever virus (CSFV) and Porcine Reproductive and Respiratory Syndrome Virus (PRRSV) attenuated live vaccine strains were purchased from Pulike Biological Inc., China, and Porcine epidemic diarrhea virus (PEDV) attenuated live vaccine strain was bought from CAHIC Inc., China.

### Sample processing, viral RNA/DNA extraction and reverse transcription

Aliquots of 0.5 g of fecal material were re-suspended in 1 mL of ice-cold phosphate-buffered saline (PBS), vortexed for 1 min, and clarified by centrifugation at 12,000 x g for 5 min. The supernatant was transferred into a 2-mL Eppendorf tube used for viral RNA extraction and the remaining was stored at -80 ^о^C until use. For RNA viruses, 0.2 mL of the supernatant of virus strains and clinical samples was used for viral RNA extraction (Tiandz Inc., Beijing, China). The viral cDNA was synthesized using the RevertAid First Strand cDNA synthesis Kit (Thermo Scientific, Waltham, MA, USA) according to the manufacturer’s instructions. All RNA extraction procedures of clinical samples included positive (PSV-HuN1 strain) and negative control (containing only water) in each run. For DNA viruses, viral DNA was carried out using the viral DNA extraction kit (Tiandz) following the manufacturer’s instructions.

### Development of a real-time PCR for PSV

To ensure the conservative of primers, the known genomic sequences of PSV were retrieved from GenBank (Table S1, up to July 2020) and analyzed with the Lasergene package (DNAStar Inc., Madison, WI, USA). After multiple alignments, a pair of primers (PSV-rF/PSV-rR), located in the conserved 5’ untranslated region, was designed to amplify a 270-bp fragment of PSV DNA (Table 3). Selected positive PCR products, amplified from cDNA of PSV-HuN1 strain, were separated by electrophoresis on a 0.8% agarose gel, and the target bands were excised and purified using the gel extraction kit (Tiandz). Then, the purified PCR products were cloned into the pMD19-T vector (Takara Bio Inc., Otsu, Japan), and the recombinant plasmids were transformed into Trans1-T1 Escherichia coli bacteria (TransGen, Beijing, China) and propagated according to the instructions of cloning manual. The plasmids were extracted using the plasmid extraction kit (Tiandz) following the manufacturer’s instructions, quantified with spectrophotometer (BioDrop, Cambridge, UK), and then sequenced (BioSune, Shanghai, China).

**Table 3:** Primers for PSV detection and complete *VP1* gene amplification

Primer name	Oligonucleotide sequence (5′-3′)^c^	Genome location
PSV-rF^a^	CGTGCTCCTTTGGTGATTC	211-229^d^
PSV-rR^b^	GAAAGAGTAGTAGTAGATTCC	460-480^d^
PSV-VP1-I-R	TGTRAANGANCCYCTRTCAAA	3245-3265^d^/2788-2808^e^/3207-3227^f^
PSV-VP1-E-R	CTCCARTTRTTDGCWGCRTGGTAGGG	3299-3324^d^/2842-2867^e^/3261-3286^f^
PSV-VP1-A-I-F	CCACCDGGHGCMCCNTCHACATG	2180-2202^d^
PSV-VP1-A-E-F	ACAYTDTCWTAYAAYGGRTGGGT	2126-2148^d^
PSV-VP1-B-I-F	AGTGCDKCRGAYAAYTTTGT	1765-1784^e^
PSV-VP1-B-E-F	TRTCRTAYAATGGRTGGGT	1673-1691^e^
PSV-VP1-C-I-F	ACAGTKAGYGCTKCRGACAACTTTG	2202-2226^f^
PSV-VP1-C-E-F	TTCCARCARACWGCACTDGTTGT	2142-2164^f^

^a^ F: forward primer

^b^ R: reverse primer

^c^*R*, A or G; *Y*, C or T; *W*, A or T; *N*, A, T, G, or C; *D*, A, G, or T; *K*, G or T; *H*, A, C, or T; *M*, A or C

^d^ Location of primers relative to the complete genomic sequence of PSV-JD2011 (GenBank # KF539414)

^e^ Location of primers relative to the complete genomic sequence of PSV-HuN1 (GenBank # KX354740)

^f^ Location of primers relative to the complete genomic sequence of PSV-V13 (GenBank # AF406813)

The plasmids were used as standards for the real-time PCR assay. Each reaction consisted of a total volume of 20 μL, containing 10 μL of the SYBR Green Master Mix (Thermo Scientific, Waltham, MA, USA), 1 μL of the standard plasmids, 0.5 μL of each of the two primers, and 8 μL of distilled water. Amplification and quantification reactions were performed using the ABI StepOnePlus^TM^ Real-Time PCR Systems (Applied Biosystems, Foster City, CA, USA) under universal conditions: 2 min at 50 °C, 2 min at 95 °C, 40 cycles of 15 s at 95 °C and 1 min at 60 °C. A melt curve was performed to verify the specificity of the amplified products under universal conditions: a denaturation step at 95 °C for 15 s, decreased to 60 °C for 1 min, and followed by temperature increase to 95 °C at a rate of 0.3 °C/s. A sample was considered negative if no CT was detected in 40 amplification cycles. The initial plasmid standard with 10-fold serial dilution (5.22×10^2^-5.22×10^8^ copies/μL) was generating the standard curve. Each dilution was run in triplicate.

The sensitivity of the real-time PCR was determined by testing 10-fold serial dilutions of the plasmid standards (5.22×10^1^-5.22×10^8^ copies/μL). Meanwhile, each diluted plasmid standard was used as templates for conventional PCR detection. PCR products were analyzed by agarose gel electrophoresis, and observed under ultraviolet light to determine sensitivity. The specificity of the primers was performed by testing samples positive for other common porcine viral pathogens, including CSFV, PRRSV, PRV, PEDV, PTV, and JEV.

### *VP1* Gene sequencing

As the sequences of the N-terminal flanking the *VP1* gene are hypervariable among PSVs, three different sets of forward primers were designed based on the grouping results of the phylogenetic analysis of these regions. Thus, three different nested-PCR methods, shared the same reverse primers, were used to amplifying the complete *VP1* gene sequence of all PSV strains (Table 3). Each method comprised an external and an internal primer pair. PCR amplification of cDNA was carried out using Pfu DNA polymerase (Tiandz) under the following conditions: an initial denaturation step at 94 °C for 4 min; 35 cycles of 94 °C for 30 s, 51 °C for 30 s and 68 °C for 3 min; and a final extension at 68 °C for 10 min. A nested PCR reaction was conducted by setting up a second reaction using 1 μL of the PCR products from the first PCR reaction as template. The PCR products were analyzed by agarose gel electrophoresis and then sequenced (BioSune, Shanghai, China).

### Phylogenetic analysis

After the removal of the flanking non-*VP1* ends, multiple sequence alignment of the complete *VP1* gene sequence was carried out using the MUSCLE method within the MEGA 6.06 software [22]. Phylogenetic analysis was carried out by PhyML 3.0 using maximum-likelihood (ML) methods [23]. The best-fit model of nucleotides for the dataset was determined using jModelTest 2.1.10 [24]. The general time Reversible (GTR) substitution model with the proportion of invariant sites and gamma-distributed rate heterogeneity (GTR+G+I) was used to construct the ML tree with a non-parametric bootstrap analysis with 100 replicates to determine the branch support. To characterize the genetic divergence of PSV, the distances were calculated by the uncorrected p-distance method using MEGA 6.06 software.

### Statistical analysis

Differences in PSV prevalence rates among age group were analyzed by chi-squared tests using SPSS software version 19.0 (IBM Inc., Chicago, IL, USA). The results were considered significant at *P* < 0.05.

## Supplementary Information

**Additional file 1: Supplementary Fig. 1.** Development of a SYBR Green I-based real-time PCR method for PSV detection. (a) Standard curves generated from the mean cycle threshold (CT) values obtained against the diluted plasmid standards (log 10 copy number). The correlation coefficient (R^2^) and the equation of the regression curve (Y) were calculated. Equation: y=-5.1538x+50.716; correlation coefficient: R^2^=0.9996. (b) Melting curve analysis of real-time PCR based on SYBR Green I. The Tm of PSV real-time PCR was 84.82 °C. (c) Specificity of the PSV SYBR Green I real-time PCR. Only the PSV-HuN1 strain showed a high-intensity fluorescent signal. CSFV, PRRSV, PRV, PEDV, PTV, and JEV did not show specific amplification. (d) Sensitivity of the SYBR Green I real-time PCR. The 10-fold serial dilutions of pMD19-T-PSV plasmids ranging from 5.22×10^8^-5.22×10^1^ copies/μL marked as 1-8, respectively. (e) The agarose gel electrophoresis results of conventional PCR. DNA marker of 5000 bp was used. The 10-fold serial dilutions of pMD19-T-PSV plasmids ranging from 5.22×10^8^-5.22 copies/μL in lanes 1-9, respectively; lane 10 contains the negative control.

**Additional file 2: Supplementary Table 1.** Sapelovirus strains used in the study.

## Data Availability

The coding sequences of the *VP1* gene were submitted to the GenBank database. The virus was named as YC1-11. The accession numbers of the *VP1* gene are MW411420-MW411430, respectively

## Abbreviations

RT-PCR: Reverse transcription polymerase chain reaction
PSV: Porcine sapelovirus
PRV: Pseudorabies virus
JEV: Japanese encephalitis virus
PTV: Porcine teschovirus
CSFV: Classical swine fever virus
PRRSV: Porcine Reproductive and Respiratory Syndrome Virus
PEDV: Porcine epidemic diarrhea virus
ICTV: International Committee on Taxonomy of Viruses
PBS: Phosphate-buffered saline
CT: Threshold cycle
ML: Maximum-likelihood
GTR: General time Reversible
Tm: Melting temperature
AA: Amino acid
