# Supplementary figures and images for: Characterization of porcine sapelovirus prevalent in western Jiangxi, China
Source: BMC Vet Res. 2021 Aug 14;17:273. doi: 10.1186/s12917-021-02979-7 (PMC8364068; doi:10.1186/s12917-021-02979-7)

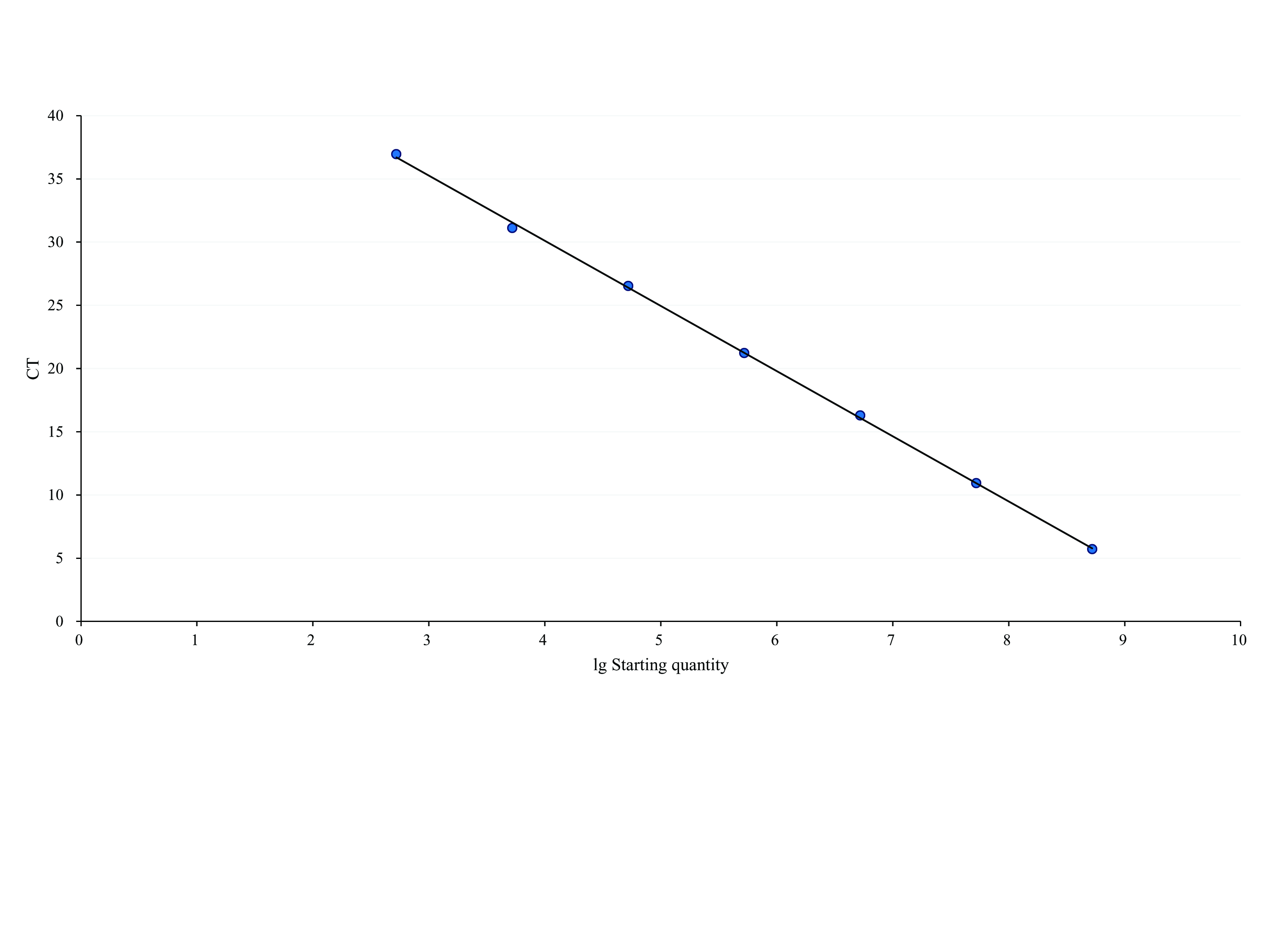

Supplement: Supplementary file 1 — Additional file 1: Supplementary Fig. 1. Development of a SYBR Green I-based real-time PCR method for PSV detection. (a) Standard curves generated from the mean cycle threshold (CT) values obtained against the diluted plasmid standards (log 10 copy number). The correlation coefficient (R2) and the equation of the regression curve (Y) were calculated. Equation: y=-5.1538x+50.716; correlation coefficient: R2=0.9996. (b) Melting curve analysis of real-time PCR based on SYBR Green I. The Tm of PSV real-time PCR was 84.82 °C. (c) Specificity of the PSV SYBR Green I real-time PCR. Only the PSV-HuN1 strain showed a high-intensity fluorescent signal. CSFV, PRRSV, PRV, PEDV, PTV, and JEV did not show specific amplification. (d) Sensitivity of the SYBR Green I real-time PCR. The 10-fold serial dilutions of pMD19-T-PSV plasmids ranging from 5.22×10^8^-5.22×101 copies/μL marked as 1-8, respectively. (e) The agarose gel electrophoresis results of conventional PCR. DNA marker of 5000 bp was used. The 10-fold serial dilutions of pMD19-T-PSV plasmids ranging from 5.22×10^8^-5.22 copies/μL in lanes 1-9, respectively; lane 10 contains the negative control. [file 12917_2021_2979_MOESM1_ESM.zip › Supplementary Fig 1a.tif]

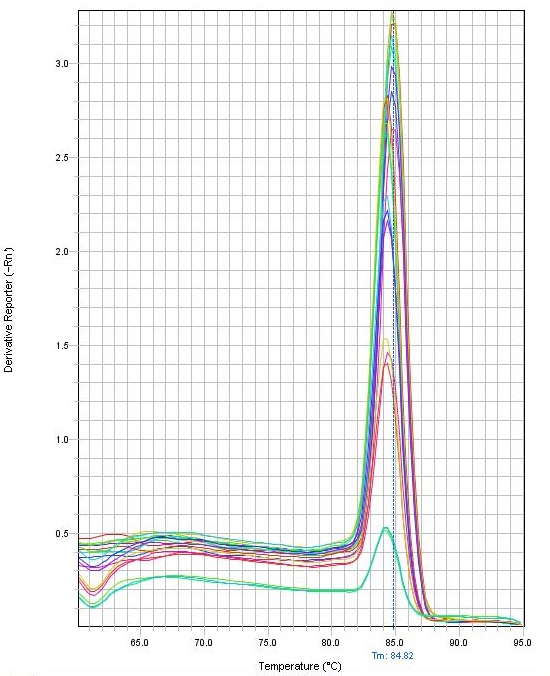

Supplement: Supplementary file 1 — Additional file 1: Supplementary Fig. 1. Development of a SYBR Green I-based real-time PCR method for PSV detection. (a) Standard curves generated from the mean cycle threshold (CT) values obtained against the diluted plasmid standards (log 10 copy number). The correlation coefficient (R2) and the equation of the regression curve (Y) were calculated. Equation: y=-5.1538x+50.716; correlation coefficient: R2=0.9996. (b) Melting curve analysis of real-time PCR based on SYBR Green I. The Tm of PSV real-time PCR was 84.82 °C. (c) Specificity of the PSV SYBR Green I real-time PCR. Only the PSV-HuN1 strain showed a high-intensity fluorescent signal. CSFV, PRRSV, PRV, PEDV, PTV, and JEV did not show specific amplification. (d) Sensitivity of the SYBR Green I real-time PCR. The 10-fold serial dilutions of pMD19-T-PSV plasmids ranging from 5.22×10^8^-5.22×101 copies/μL marked as 1-8, respectively. (e) The agarose gel electrophoresis results of conventional PCR. DNA marker of 5000 bp was used. The 10-fold serial dilutions of pMD19-T-PSV plasmids ranging from 5.22×10^8^-5.22 copies/μL in lanes 1-9, respectively; lane 10 contains the negative control. [file 12917_2021_2979_MOESM1_ESM.zip › Supplementary Fig 1b.jpg]

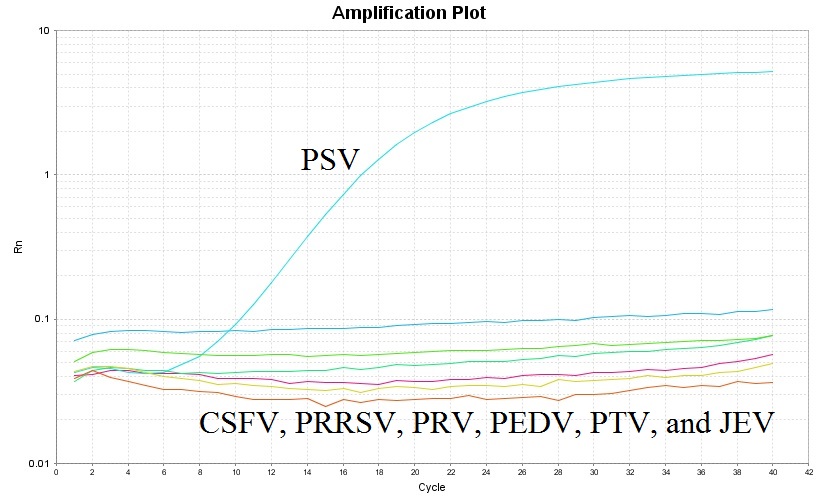

Supplement: Supplementary file 1 — Additional file 1: Supplementary Fig. 1. Development of a SYBR Green I-based real-time PCR method for PSV detection. (a) Standard curves generated from the mean cycle threshold (CT) values obtained against the diluted plasmid standards (log 10 copy number). The correlation coefficient (R2) and the equation of the regression curve (Y) were calculated. Equation: y=-5.1538x+50.716; correlation coefficient: R2=0.9996. (b) Melting curve analysis of real-time PCR based on SYBR Green I. The Tm of PSV real-time PCR was 84.82 °C. (c) Specificity of the PSV SYBR Green I real-time PCR. Only the PSV-HuN1 strain showed a high-intensity fluorescent signal. CSFV, PRRSV, PRV, PEDV, PTV, and JEV did not show specific amplification. (d) Sensitivity of the SYBR Green I real-time PCR. The 10-fold serial dilutions of pMD19-T-PSV plasmids ranging from 5.22×10^8^-5.22×101 copies/μL marked as 1-8, respectively. (e) The agarose gel electrophoresis results of conventional PCR. DNA marker of 5000 bp was used. The 10-fold serial dilutions of pMD19-T-PSV plasmids ranging from 5.22×10^8^-5.22 copies/μL in lanes 1-9, respectively; lane 10 contains the negative control. [file 12917_2021_2979_MOESM1_ESM.zip › Supplementary Fig 1c.jpg]

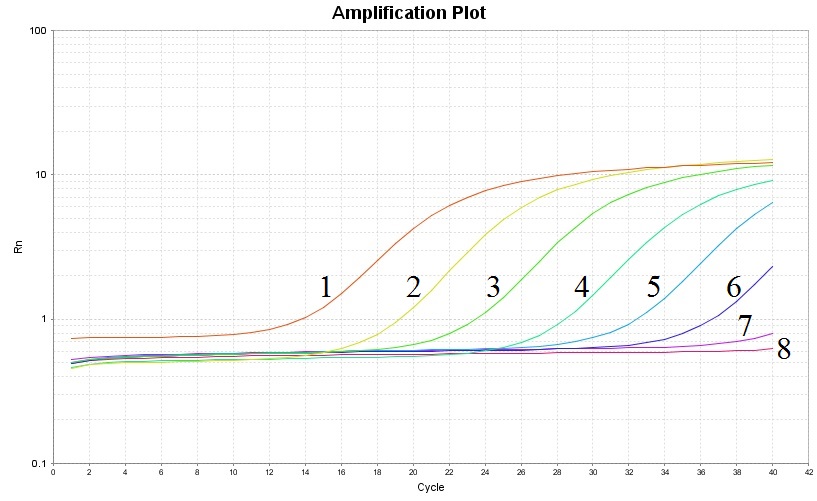

Supplement: Supplementary file 1 — Additional file 1: Supplementary Fig. 1. Development of a SYBR Green I-based real-time PCR method for PSV detection. (a) Standard curves generated from the mean cycle threshold (CT) values obtained against the diluted plasmid standards (log 10 copy number). The correlation coefficient (R2) and the equation of the regression curve (Y) were calculated. Equation: y=-5.1538x+50.716; correlation coefficient: R2=0.9996. (b) Melting curve analysis of real-time PCR based on SYBR Green I. The Tm of PSV real-time PCR was 84.82 °C. (c) Specificity of the PSV SYBR Green I real-time PCR. Only the PSV-HuN1 strain showed a high-intensity fluorescent signal. CSFV, PRRSV, PRV, PEDV, PTV, and JEV did not show specific amplification. (d) Sensitivity of the SYBR Green I real-time PCR. The 10-fold serial dilutions of pMD19-T-PSV plasmids ranging from 5.22×10^8^-5.22×101 copies/μL marked as 1-8, respectively. (e) The agarose gel electrophoresis results of conventional PCR. DNA marker of 5000 bp was used. The 10-fold serial dilutions of pMD19-T-PSV plasmids ranging from 5.22×10^8^-5.22 copies/μL in lanes 1-9, respectively; lane 10 contains the negative control. [file 12917_2021_2979_MOESM1_ESM.zip › Supplementary Fig 1d.jpg]

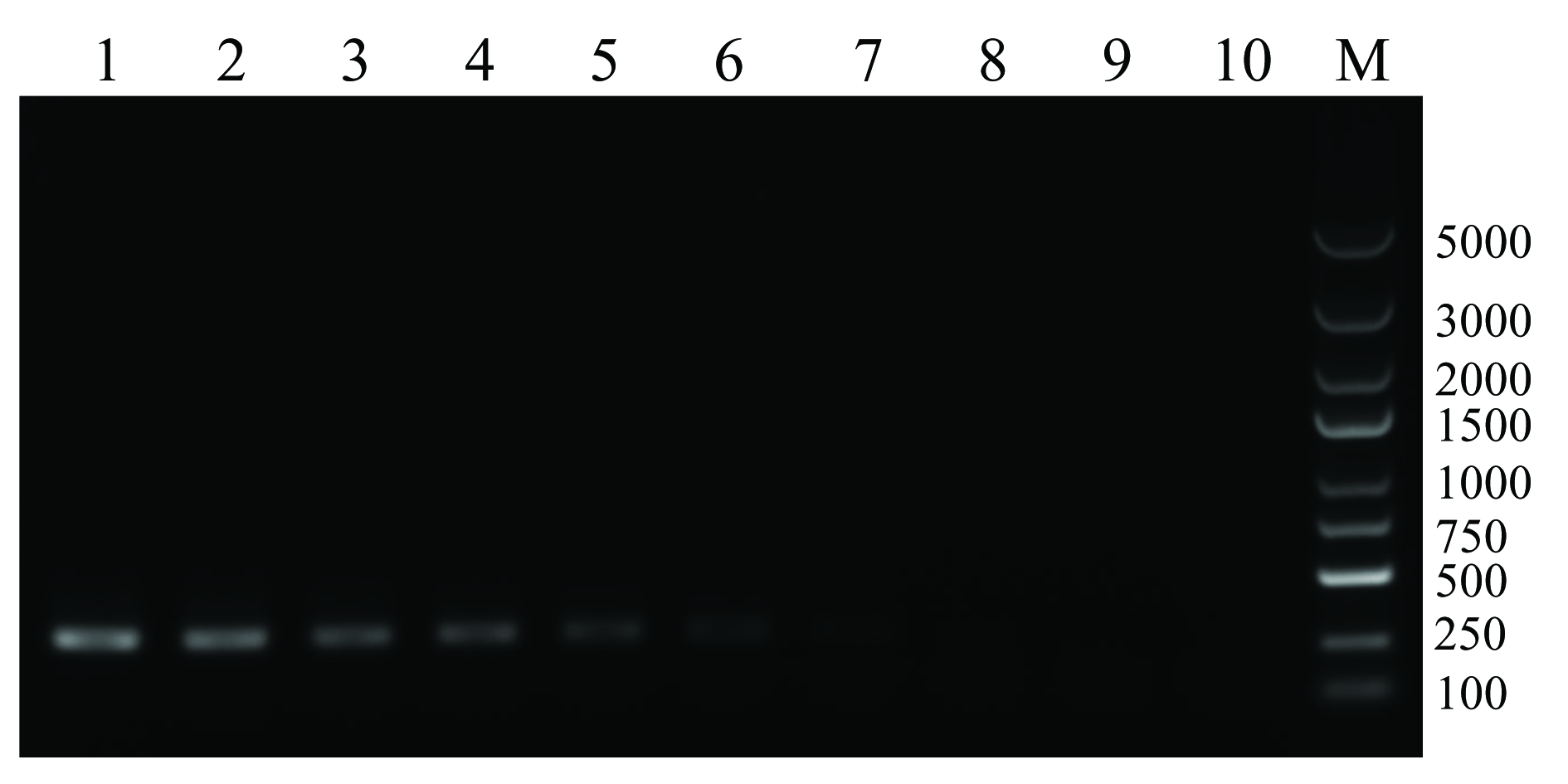

Supplement: Supplementary file 1 — Additional file 1: Supplementary Fig. 1. Development of a SYBR Green I-based real-time PCR method for PSV detection. (a) Standard curves generated from the mean cycle threshold (CT) values obtained against the diluted plasmid standards (log 10 copy number). The correlation coefficient (R2) and the equation of the regression curve (Y) were calculated. Equation: y=-5.1538x+50.716; correlation coefficient: R2=0.9996. (b) Melting curve analysis of real-time PCR based on SYBR Green I. The Tm of PSV real-time PCR was 84.82 °C. (c) Specificity of the PSV SYBR Green I real-time PCR. Only the PSV-HuN1 strain showed a high-intensity fluorescent signal. CSFV, PRRSV, PRV, PEDV, PTV, and JEV did not show specific amplification. (d) Sensitivity of the SYBR Green I real-time PCR. The 10-fold serial dilutions of pMD19-T-PSV plasmids ranging from 5.22×10^8^-5.22×101 copies/μL marked as 1-8, respectively. (e) The agarose gel electrophoresis results of conventional PCR. DNA marker of 5000 bp was used. The 10-fold serial dilutions of pMD19-T-PSV plasmids ranging from 5.22×10^8^-5.22 copies/μL in lanes 1-9, respectively; lane 10 contains the negative control. [file 12917_2021_2979_MOESM1_ESM.zip › Supplementary Fig 1e.tif]

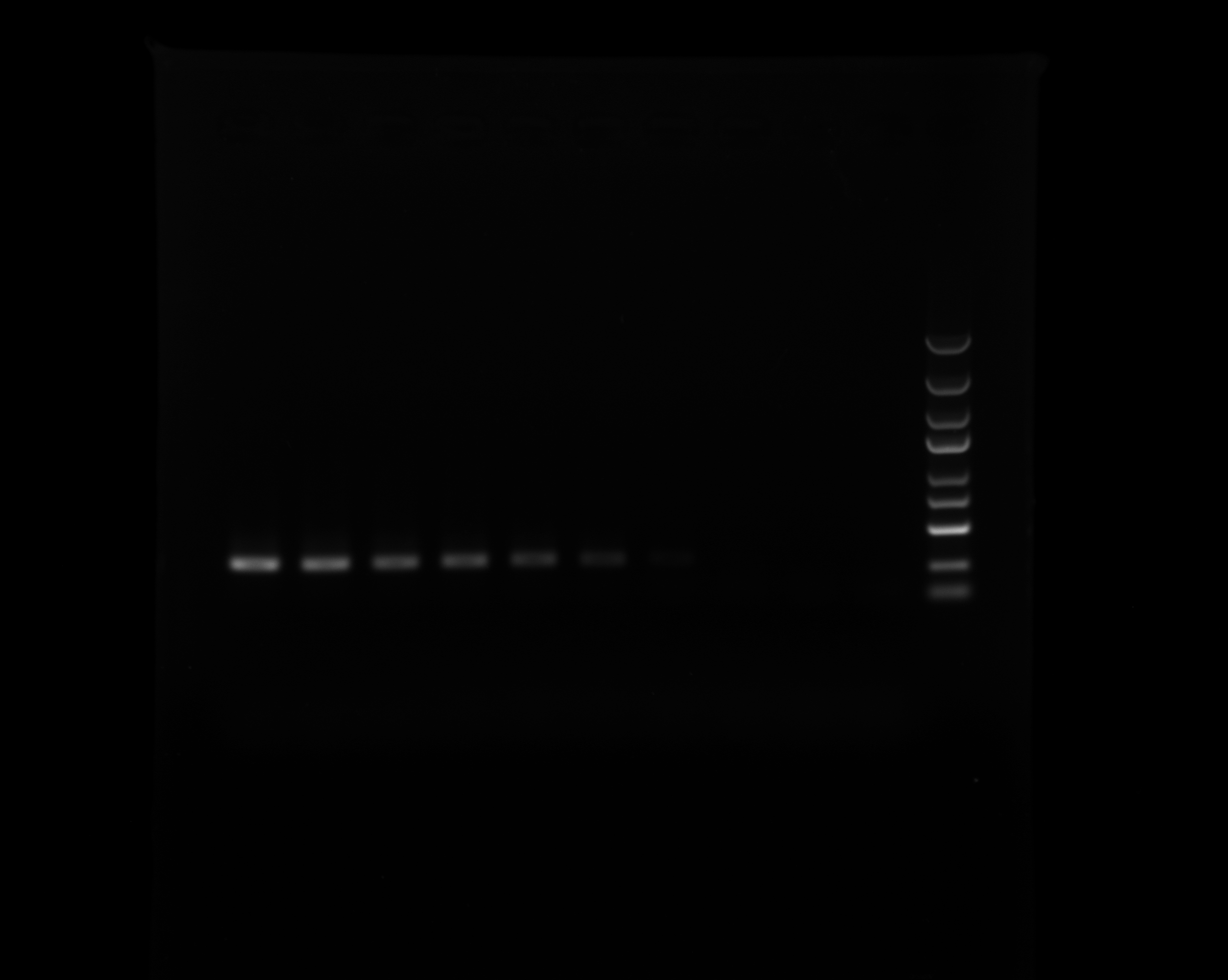

Supplement: Supplementary file 1 — Additional file 1: Supplementary Fig. 1. Development of a SYBR Green I-based real-time PCR method for PSV detection. (a) Standard curves generated from the mean cycle threshold (CT) values obtained against the diluted plasmid standards (log 10 copy number). The correlation coefficient (R2) and the equation of the regression curve (Y) were calculated. Equation: y=-5.1538x+50.716; correlation coefficient: R2=0.9996. (b) Melting curve analysis of real-time PCR based on SYBR Green I. The Tm of PSV real-time PCR was 84.82 °C. (c) Specificity of the PSV SYBR Green I real-time PCR. Only the PSV-HuN1 strain showed a high-intensity fluorescent signal. CSFV, PRRSV, PRV, PEDV, PTV, and JEV did not show specific amplification. (d) Sensitivity of the SYBR Green I real-time PCR. The 10-fold serial dilutions of pMD19-T-PSV plasmids ranging from 5.22×10^8^-5.22×101 copies/μL marked as 1-8, respectively. (e) The agarose gel electrophoresis results of conventional PCR. DNA marker of 5000 bp was used. The 10-fold serial dilutions of pMD19-T-PSV plasmids ranging from 5.22×10^8^-5.22 copies/μL in lanes 1-9, respectively; lane 10 contains the negative control. [file 12917_2021_2979_MOESM1_ESM.zip › Supplementary Fig 1e_original version.tif]
