# Supplementary material for: Characterization of porcine sapelovirus prevalent in western Jiangxi, China
Source: BMC Vet Res. 2021 Aug 14;17:273. doi: 10.1186/s12917-021-02979-7 (PMC8364068; doi:10.1186/s12917-021-02979-7)
Supplement: Supplementary file 2 — Additional file 2: Supplementary Table 1. Sapelovirus strains used in the study. [file 12917_2021_2979_MOESM2_ESM.docx]

Supplementary Table 1 Sapelovirus strains used in the study.

| Virus isolate | Collection date | GenBank accession No. | Geographic origin | Sequence length |
| --- | --- | --- | --- | --- |
| HuN1 | 2015 | KX354740 | China | 6996 |
| HuN2 | 2015 | KX354741 | China | 6996 |
| HuN29 | 2017 | MF440657 | China | 7220 |
| HuN30 | 2017 | MF440658 | China | 7259 |
| HuN20 | 2017 | MF440648 | China | 7223 |
| HuN27 | 2017 | MF440655 | China | 7263 |
| HuN13 | 2016 | MF440641 | China | 7254 |
| HuN14 | 2016 | MF440642 | China | 7152 |
| HuN11 | 2016 | MF440639 | China | 7231 |
| HuN16 | 2016 | MF440644 | China | 7227 |
| HuN8 | 2016 | MF440636 | China | 7222 |
| HuN7 | 2016 | MF440635 | China | 7264 |
| HuN18 | 2016 | MF440646 | China | 7225 |
| HuN5 | 2016 | MF440633 | China | 7218 |
| HuN24 | 2017 | MF440652 | China | 7221 |
| HuN15 | 2016 | MF440643 | China | 7221 |
| QT2013 | 2013 | KJ463384 | China | 7532 |
| HuN33 | 2014 | MF440661 | China | 7222 |
| NM02_C1 | 2017 | MK378953 | China | 7458 |
| HuN31 | 2017 | MF440659 | China | 7232 |
| HuN6 | 2016 | MF440634 | China | 7210 |
| HeB04 | 2017 | MK378925 | China | 7382 |
| XTND/2018 | 2018 | LC493088 | Viet Nam | 7537 |
| HuN25 | 2014 | MF440653 | China | 7254 |
| YN02 | 2017 | MK378966 | China | 7347 |
| csh | 2009 | HQ875059 | China | 7502 |
| HuN4 | 2015 | KX354743 | China | 6999 |
| JD2011 | 2011 | KF539414 | China | 7572 |
| YC2011 | 2011 | JX286666 | China | 7572 |
| PSV-20-V | 2018 | LC508226 | Zambia | 7495 |
| HuN23 | 2017 | MF440651 | China | 7221 |
| HuN3 | 2015 | KX354742 | China | 6996 |
| JXXY-C | 2017 | MH626635 | China | 7575 |
| PSV/Porcine/JPN/MoI2/2016 | 2016 | LC425414 | Japan | 7514 |
| SHCM2019 | 2019 | MN685785 | China | 7567 |
| HuN32 | 2017 | MF440660 | China | 7227 |
| PSV/Porcine/JPN/HgOg11/2018 | 2018 | LC425417 | Japan | 7521 |
| PSV-23-V | 2018 | LC508230 | Zambia | 7462 |
| PSV/Porcine/JPN/MoI3/2016 | 2016 | LC425415 | Japan | 7540 |
| PSV/Porcine/JPN/MoI2-2/2015 | 2015 | LC425394 | Japan | 7425 |
| PSV-26-B | 2018 | LC508232 | Zambia | 7493 |
| PSV/Porcine/JPN/HgTa2-2/2015 | 2015 | LC425404 | Japan | 7525 |
| PSV/Porcine/JPN/HgOg2-5/2015 | 2015 | LC425395 | Japan | 7480 |
| HuN12 | 2016 | MF440640 | China | 7227 |
| HuN9 | 2016 | MF440637 | China | 7245 |
| PSV/Porcine/JPN/HgYa1/2016 | 2016 | LC425413 | Japan | 7526 |
| PSV/Porcine/JPN/Iba26-489S/2015 | 2014 | LC425397 | Japan | 7531 |
| KS055217 | 2005 | KJ821021 | South Korea | 7542 |
| KS05151 | 2005 | KJ821020 | South Korea | 7566 |
| PSV/Porcine/JPN/HkKa2-3/2015 | 2015 | LC425403 | Japan | 7540 |
| KS04105 | 2004 | KJ821019 | South Korea | 7542 |
| PSV-46-V | 2018 | LC508233 | Zambia | 7469 |
| PSV/Porcine/JPN/HgYa2-3/2015 | 2015 | LC425407 | Japan | 7535 |
| PSV/Porcine/JPN/HgTa2-1/2015 | 2015 | LC425401 | Japan | 7540 |
| USA/IA33375/2015 | 2015 | KX574284 | USA | 7565 |
| PSV/Porcine/JPN/HgYa2-1/2015 | 2015 | LC425405 | Japan | 7525 |
| PSV/Porcine/JPN/DeTk2-2/2015 | 2015 | LC425396 | Japan | 7428 |
| PSV/Porcine/JPN/Ishi-Ka2/2015 | 2015 | LC425398 | Japan | 7543 |
| Jpsv447 | 2009 | LC326556 | Japan | 7582 |
| PSV/Porcine/JPN/Ishi-Ka2/2017 | 2017 | LC425416 | Japan | 7534 |
| PSV/Porcine/JPN/Ishi-Im3/2015 | 2015 | LC425410 | Japan | 7554 |
| PSV/Porcine/JPN/Ishi-Miya3/2015 | 2015 | LC425399 | Japan | 7508 |
| ISU-SHIC | 2016 | KX810827 | USA | 7389 |
| PSV/Porcine/JPN/Ishi-Ya8/2015 | 2015 | LC425412 | Japan | 7528 |
| IVRI/PSV/SPF/C-6/2015 | 2015 | KY053835 | India | 7491 |
| V13 | 1960 | AF406813 | Germany | 7491 |
| OPY-1-Corsica-2017 | 2017 | MH513612 | France | 7532 |
| DIAPD5469-10 | 2015 | MK497044 | Italy | 7564 |
| PSV_GER_L00798-K11_14-02_2014 | 2014 | LT900497 | Germany | 7499 |
| SZ1M-F/PSV/HUN/2013 | 2013 | MN807752 | Hungary | 7534 |
| SwPSV75BO2012 | 2012 | MN836683 | Italy | 7560 |
| GX03_C1 | 2017 | MK378906 | China | 7263 |
| GX04_C1 | 2017 | MK378907 | China | 7300 |
| HLJ01_C1 | 2017 | MK378928 | China | 6445 |
| GZ04_C1 | 2017 | MK378918 | China | 6829 |
| ZJ01_C1 | 2017 | MK378967 | China | 7133 |
| AH01_C1 | 2017 | MK378881 | China | 7191 |
| GX01_C1 | 2017 | MK378899 | China | 4967 |
| Sek_1562/98 | 1998 | AY392556 | Germany | 3968 |
| Po_5116 | 1998 | AY392538 | Germany | 3969 |
| 26-T-XII | 1998 | AY392544 | Germany | 3688 |
| 16-S-X | 1998 | AY392543 | Germany | 3699 |
| EF9-F/PSV/HUN/2016 | 2016 | MN807773 | Hungary | 854 |
| JS/CHN/2016 | 2016 | MH422121 | China | 855 |
| SD/CHN/2016 | 2015 | MH422124 | China | 855 |
| NMG02/CHN/2016 | 2016 | MH422125 | China | 855 |
| A5-RS/PSV/HUN/2016 | 2016 | MN807776 | Hungary | 879 |
| A1-RS/PSV/HUN/2016 | 2016 | MN807754 | Hungary | 879 |
| GD1-RS/PSV/HUN/2016 | 2016 | MN807768 | Hungary | 879 |
| A3-RS/PSV/HUN/2016 | 2016 | MN807756 | Hungary | 867 |
| P1-3-3 | 2007 | KF705632 | Spain | 1171 |
| TM1121-F/PSV/HUN/2013 | 2013 | MN807769 | Hungary | 879 |
| SZ4M-F/PSV/HUN/2013 | 2013 | MN807753 | Hungary | 867 |
| B4-RS/PSV/HUN/2016 | 2016 | MN807758 | Hungary | 879 |
| BUV1-F/PSV/HUN/2013 | 2013 | MN807759 | Hungary | 879 |
| D4-NS/PSV/HUN/2016 | 2016 | MN807764 | Hungary | 855 |
| Gifu | - | AB619806 | Japan | 855 |
| ZS2-F/PSV/HUN/2013 | 2013 | MN807771 | Hungary | 855 |
| EF2-F/PSV/HUN/2016 | 2016 | MN807765 | Hungary | 879 |
| VC4-EF2-F/PSV/HUN/2017 | 2017 | MN807777 | Hungary | 879 |
| HuN/CHN/2016 | 2016 | MH422123 | China | 855 |
| 2383 | - | AY064708 | USA | 8126 |
| VRDL1 | - | EU789367 | USA | 7971 |
| WUHARV | 2010 | JX627573 | USA | 8059 |

-, Not identified.
